# Supplementary material for: Nuclear soluble cGAS senses double-stranded DNA virus infection
Source: Commun Biol. 2022 May 10;5:433. doi: 10.1038/s42003-022-03400-1 (PMC9090744; doi:10.1038/s42003-022-03400-1)
Supplement: Supplementary file 2 — Supplementary Information [file 42003_2022_3400_MOESM2_ESM.pdf]

Supplemental Figure Legends

Supplemental Figure 1. Endogenous cGAS localizes in the nucleus and tethers to the chromatin. (**a**) THP-1 cells were transfected with or without 1 μg/mL ctDNA for 4 h. Then, the cell lysates were fractionated into five fractions and blotted as indicated. STING: membrane marker; TBK1 and β-actin: cytosolic marker; H3: nuclear marker; vimentin: cytoskeletal marker. (**b**) Lysates of cGAS wild type (WT) and knockout (KO) H1299 cells were blotted as indicated. (**c-f**) IFA of endogenous cGAS in THP-1 cells (**c**), HFF-1 cells (**d**), H596 cells (**e**), and MDA-MB-231 cells (**f**) transfected with or without ctDNA. cGAS: green; DAPI, blue. Bar = 10 μm. (**g**) Schematics of cGAS domains, mutants, nuclear export signal (NES), and mitochondrial targeting signal (MTS). (**h**) Summary of the subcellular localization of FLAG-tagged human cGAS (hcGAS) and the NES deletion mutant (hcGAS-delNES) transfected in HEK293 cells. cGAS localization was determined by IFA using the anti-FLAG antibody. C: cytosolic; N: nuclear; C+N: cytosolic and nuclear. Data represent means ± s.d. of three independent experiments (> 200 cells were counted in each field and five fields were counted per experiment). (**i**) HEK293 cells stably expressing hcGAS-N or hcGAS-delN were collected and fractionated into five fractions. The fractions were blotted as indicated. C1QBP: membrane marker; α-tubulin: cytosolic marker; H3: nuclear marker.

Supplemental Figure 2. HSV-1 infection induces nuclear soluble cGAS. (**a**) RAW 264.7 macrophages were infected with 1 MOI of HSV-1 KOS for 16 h, and then the subcellular fractions of cell lysates were blotted as indicated. (**b**) RAW264.7 macrophages were transfected with or without 1 μg/mL ctDNA for 4 h. Then, the cell lysates were fractionated into five fractions and blotted as indicated. (**c**) THP-1 monocytes were infected with 1 MOI of HSV-1 McKrae for 16 h, and then the subcellular fractions of cell lysates were blotted as indicated. (**d**) Mouse bone marrow-derived macrophages were infected with 1 MOI of HSV-1 McKrae for 16 h, and then the subcellular fractions of cell lysates were blotted as indicated. STING: membrane marker; α-tubulin:

cytosolic marker; H3: nuclear marker; ICP8: HSV-1 viral protein. The arrow indicates nuclear soluble cGAS after viral infection.

Supplemental Figure 3. Nuclear soluble cGAS is constitutively active. (**a**) HEK293 cells stably expressing mcGAS or the mcGAS R241E mutant were fractionated and blotted as indicated. C1QBP: membrane marker; α-tubulin: cytosolic marker; H3: nuclear marker. The arrow indicates nuclear soluble cGAS. (**b**) Wild type HEK293 cells, HEK293 cells stably expressing mcGAS or the R241E mutant were lysed. The lysates were harvested for *in vitro* cGAS enzymatic activity assays with vs. without adding ctDNA into the enzymatic reaction mixture. The production of cGAMP was determined by ELISA. Data represent means ± s.d. of three independent experiments. The *P*-value was calculated by two-way ANOVA followed by Sidak's multiple comparisons test. (**c**) RAW264.7 cells were mock transfected, transfected with ctDNA or infected with HSV-1 McKrae for 16 h. The cytoplasmic, nuclear soluble and chromatin-bound fractions were harvested and then each fraction was added ctDNA for *in vitro* cGAS enzymatic activity assays. Data represent means ± s.d. of three independent experiments. The *P*-value was calculated by two-way ANOVA followed by Sidak's multiple comparisons test. (**d**) RAW264.7 cells were mock infected or infected with HSV-1, VACV, IAV, or VSV for 16 h. The nuclear soluble extracts were harvested and then added ctDNA for *in vitro* cGAS enzymatic activity assays. Data represent means ± s.d. of three independent experiments. The *P*-value was calculated by one-way ANOVA followed by Dunnett's multiple comparisons test.

Supplemental Figure 4. Establishment of doxycycline-induced cGAS stable lines. (**a**) Lysates of wild type HEK293 and HEK293 stably expressing doxycycline-induced mcGAS or indicated mcGAS mutants were blotted as indicated. (**b**) HEK293 cells stably expressing Dox-inducible, FLAG-tagged mcGAS, mcGAS-NLS, or LL/RK-NLS were treated with 2 μg/mL Dox for

24 h. Then, the cells were fixed and stained with anti-cGAS antibody and DAPI. FLAG: red; DAPI, blue. Bar = 10 μm. (**c**) Lysates of cGAS wild type and knockout RAW264.7 macrophages were blotted as indicated. (**d**) Wild type and cGAS knockout RAW264.7 macrophages were mock infected or infected with HSV-1 d109 for 16 h. Real-time PCR was performed to determine the relative mRNA levels of IFNβ, IP-10, and RANTES. Data represent means ± s.d. of three independent experiments. The *P*-value was calculated by two-way ANOVA followed by Sidak's multiple comparisons test. (**e**) Lysates of RAW264.7 cells, cGAS KO cells, KO cells reconstituted with mcGAS and LL/RK-NLS cells were blotted as indicated.

Supplemental Figure 5. Nuclear soluble cGAS induces innate immune responses. (**a**) The cGAS KO, KO(mcGAS), KO(LL/RK-NLS) RAW264.7 cells were treated with Dox for 24 h and then infected with 1 MOI of HSV-1 KOS d109 for designated times. Real-time PCR was performed to determine the relative RANTES mRNA levels. Data represent means ± s.d. of three independent experiments. The *P*-value was calculated by two-way ANOVA followed by Dunnett's multiple comparisons test. (**b**) The cGAS KO, KO(mcGAS), KO(LL/RK-NLS) RAW264.7 cells were treated with Dox for 24 h and then stimulated with 1 μg/mL of ctDNA by transfection for designated times. Real-time PCR was performed to determine the relative IP-10 mRNA levels. Data represent means ± s.d. of three independent experiments. The *P*-value was calculated by two-way ANOVA followed by Tukey’s multiple comparisons test. (**c**) The doxycycline-induced LL/RK-NLS mutant expressing HEK293 and HEK293T cells were treated with Dox for 24 h. Cell lysates were harvested and blotted as indicated. (**d**) The doxycycline-induced LL/RK-NLS mutant expressing HEK293 and HEK293T cells were treated with Dox for 24 h and then infected with 1 MOI of HSV-1 d109 for 16 h. Cell lysates were harvested and blotted as indicated. (**e**) The doxycycline-induced LL/RK-NLS mutant expressing HEK293 and HEK293T cells were treated with Dox for 24 h and then infected with 1 MOI of HSV-1 KOS d109 for 16 h. Real-time PCR was

performed to determine the relative IFNβ mRNA levels. Data represent means ± s.d. of three independent experiments. The *P*-value was calculated by two-way ANOVA followed by Sidak's multiple comparisons test. (**f**) HEK293 cells stably expressing FLAG tagged LL/RK-NLS mutant were infected with HSV-1-GFP for 16 h. ChIP assays were performed using the anti-FLAG antibody. Real-time PCR was performed to determine the relative binding amount of GFP and viral gene VP16. Data represent means ± s.d. of three independent experiments. The *P*-value was calculated by two-way ANOVA followed by Sidak's multiple comparisons test.

Supplemental Figure 6. cGAS limits VACV and VSV infection. (**a**) The cGAS WT and KO RAW264.7 macrophages were infected with VACV-Luc and VSV-Luc for 16 h. Luciferase activities were measured to determine the relative infection activity. Data represent means ± s.d. of three independent experiments. The *P*-value was calculated by two-way ANOVA followed by Sidak's multiple comparisons test.

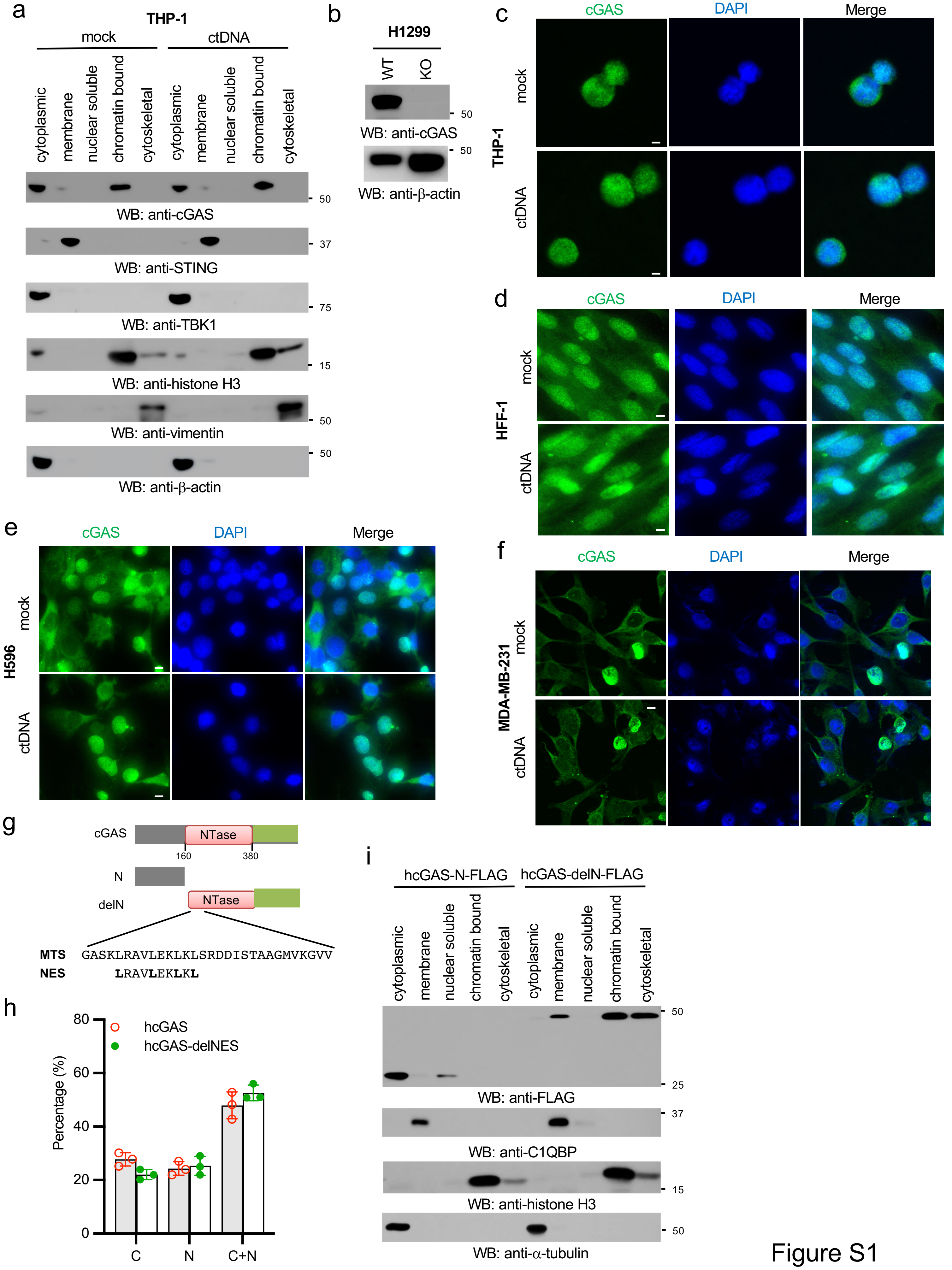
a
THP-1
mock
ctDNA
cytoplasmic
membrane
nuclear soluble
chromatin bound
cytoskeletal
WB: anti-cGAS
WB: anti-STING
WB: anti-TBK1
WB: anti-histone H3
WB: anti-vimentin
WB: anti-β-actin
b
H1299
WT
KO
WB: anti-cGAS
WB: anti-β-actin
c
cGAS
DAPI
Merge
THP-1
d
HFF-1
e
H596
f
MDA-MB-231
g
cGAS
NTase
160
380
N
delN
MTS GASKLRAVLEKLKLSRDDISTAAGMVKGVV
NES LRAVLEKLKL
h
Percentage (%)
hcGAS
hcGAS-delNES
C
N
C+N
i
hcGAS-N-FLAG
hcGAS-delN-FLAG
WB: anti-FLAG
WB: anti-C1QBP
WB: anti-histone H3
WB: anti-α-tubulin

Figure S1

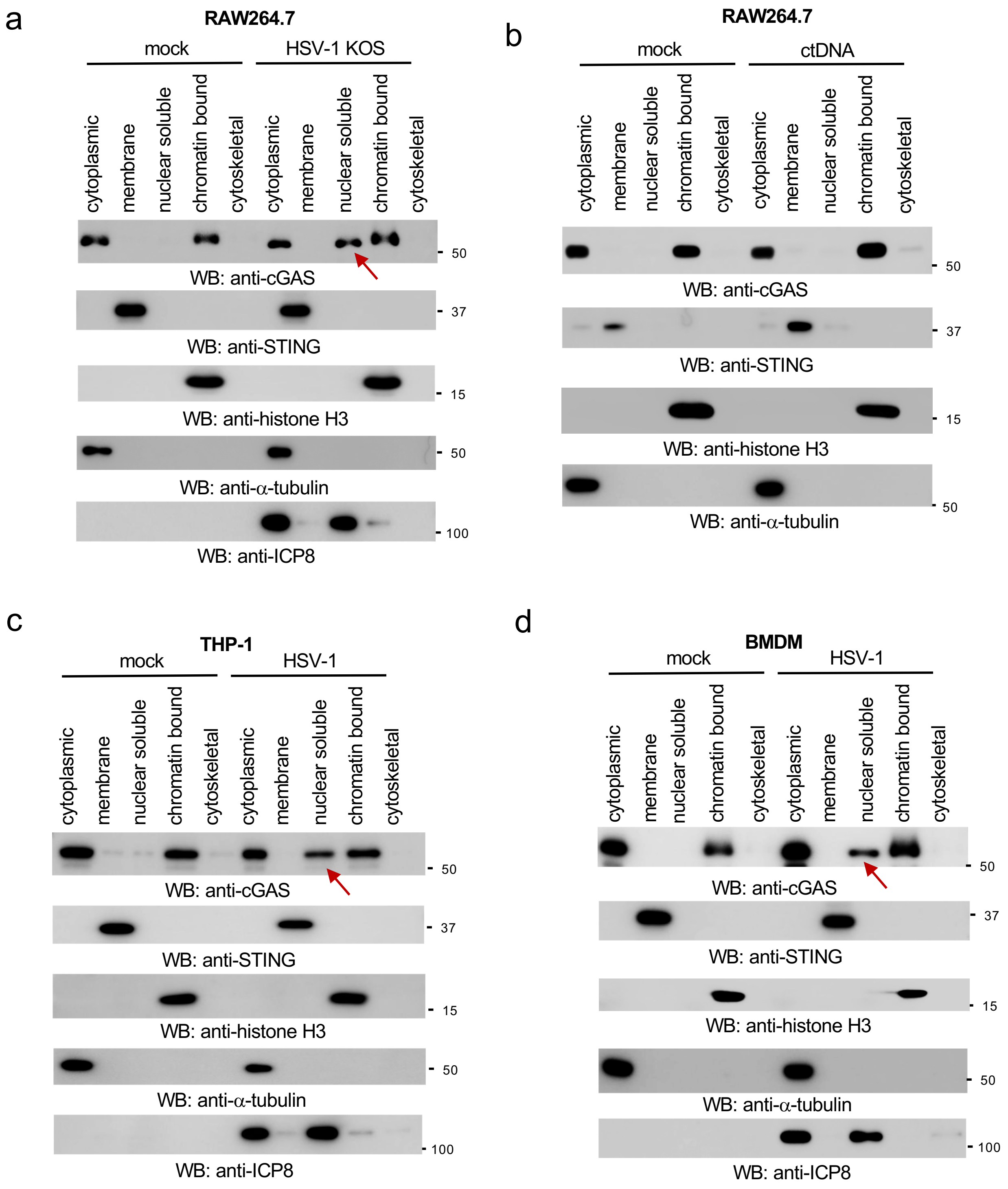
a
RAW264.7
mock
HSV-1 KOS
cytoplasmic
membrane
nuclear soluble
chromatin bound
cytoskeletal
cytoplasmic
membrane
nuclear soluble
chromatin bound
cytoskeletal
50
WB: anti-cGAS
37
WB: anti-STING
15
WB: anti-histone H3
50
WB: anti-α-tubulin
100
WB: anti-ICP8
b
RAW264.7
mock
ctDNA
cytoplasmic
membrane
nuclear soluble
chromatin bound
cytoskeletal
cytoplasmic
membrane
nuclear soluble
chromatin bound
cytoskeletal
50
WB: anti-cGAS
37
WB: anti-STING
15
WB: anti-histone H3
50
WB: anti-α-tubulin
c
THP-1
mock
HSV-1
cytoplasmic
membrane
nuclear soluble
chromatin bound
cytoskeletal
cytoplasmic
membrane
nuclear soluble
chromatin bound
cytoskeletal
50
WB: anti-cGAS
37
WB: anti-STING
15
WB: anti-histone H3
50
WB: anti-α-tubulin
100
WB: anti-ICP8
d
BMDM
mock
HSV-1
cytoplasmic
membrane
nuclear soluble
chromatin bound
cytoskeletal
cytoplasmic
membrane
nuclear soluble
chromatin bound
cytoskeletal
50
WB: anti-cGAS
37
WB: anti-STING
15
WB: anti-histone H3
50
WB: anti-α-tubulin
100
WB: anti-ICP8

Figure S2

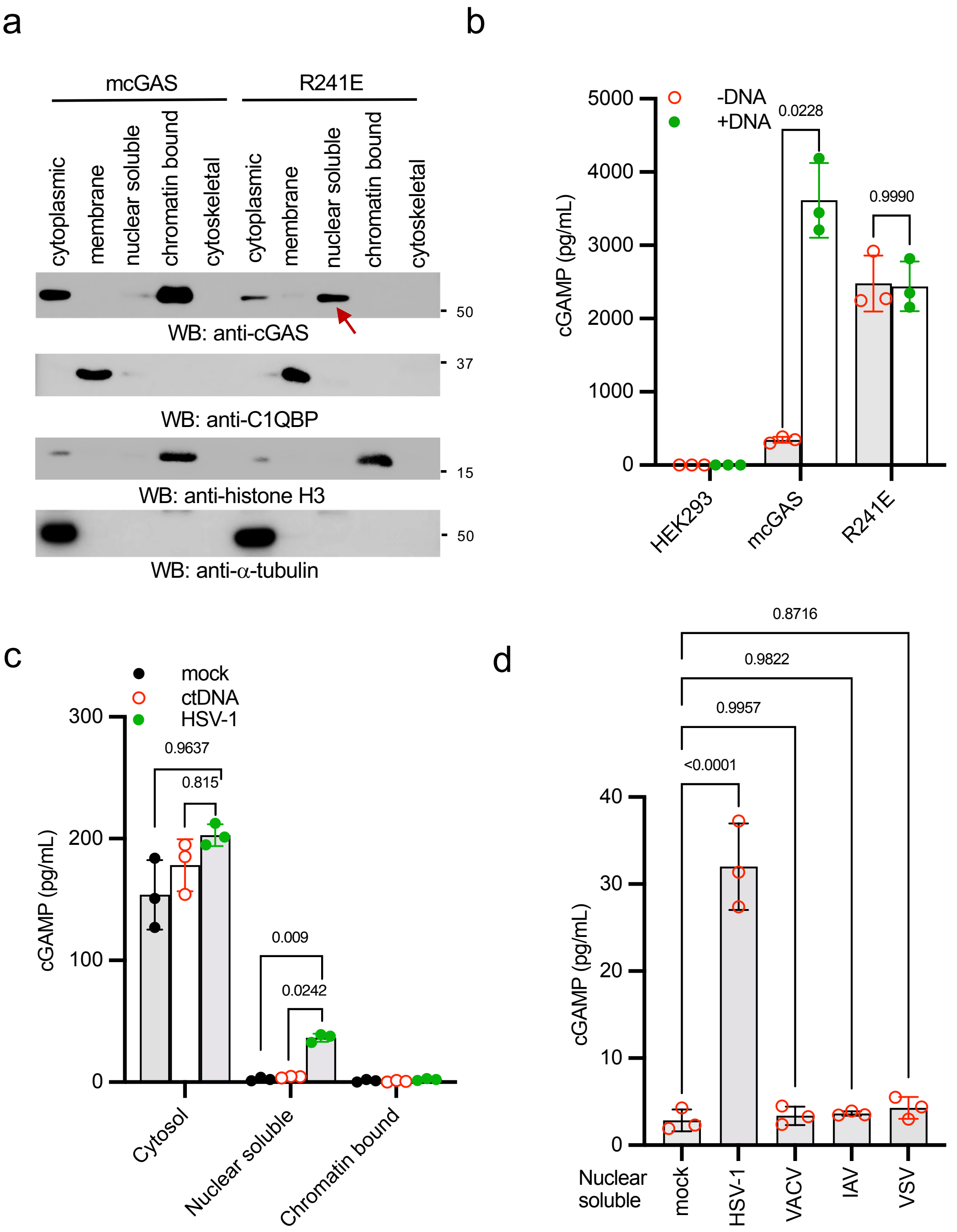
a
mcGAS
R241E
cytoplasmic
membrane
nuclear soluble
chromatin bound
cytoskeletal
50
WB: anti-cGAS
37
WB: anti-C1QBP
15
WB: anti-histone H3
50
WB: anti-α-tubulin
b
-DNA
+DNA
0.0228
0.9990
cGAMP (pg/mL)
5000
4000
3000
2000
1000
0
HEK293
mcGAS
R241E
c
mock
ctDNA
HSV-1
300
200
100
0
0.9637
0.815
0.009
0.0242
cGAMP (pg/mL)
Cytosol
Nuclear soluble
Chromatin bound
d
0.8716
0.9822
0.9957
<0.0001
40
30
20
10
0
cGAMP (pg/mL)
Nuclear soluble
mock
HSV-1
VACV
IAV
VSV

Figure S3

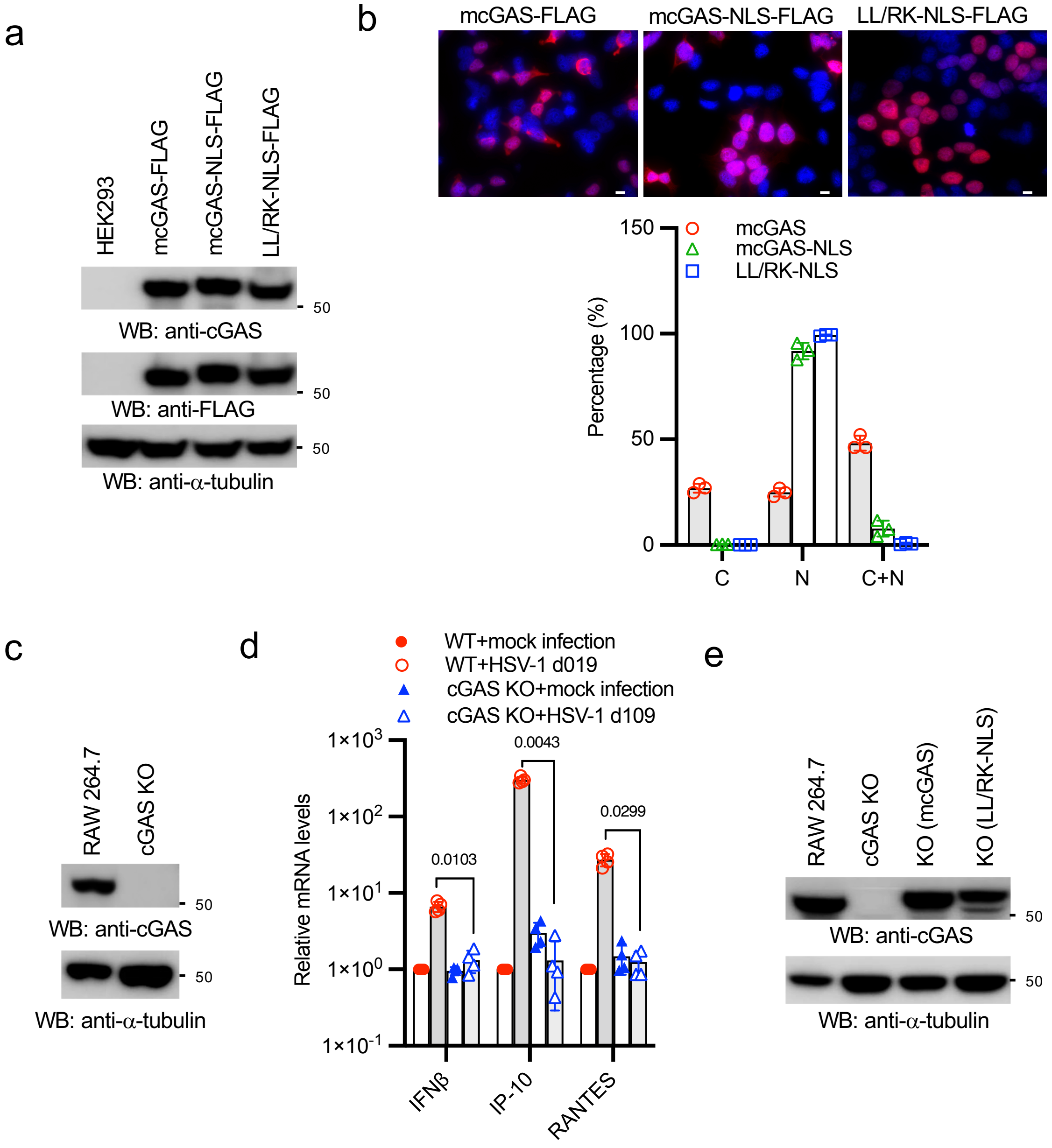
a
HEK293
mcGAS-FLAG
mcGAS-NLS-FLAG
LL/RK-NLS-FLAG
50
WB: anti-cGAS
50
WB: anti-FLAG
50
WB: anti-α-tubulin
b
mcGAS-FLAG
mcGAS-NLS-FLAG
LL/RK-NLS-FLAG
mcGAS
mcGAS-NLS
LL/RK-NLS
Percentage (%)
150
100
50
0
C
N
C+N
c
RAW 264.7
cGAS KO
50
WB: anti-cGAS
50
WB: anti-α-tubulin
d
WT+mock infection
WT+HSV-1 d019
cGAS KO+mock infection
cGAS KO+HSV-1 d109
Relative mRNA levels
1×10^3
1×10^2
1×10^1
1×10^0
1×10^-1
0.0103
0.0043
0.0299
IFNβ
IP-10
RANTES
e
RAW 264.7
cGAS KO
KO (mcGAS)
KO (LL/RK-NLS)
50
WB: anti-cGAS
50
WB: anti-α-tubulin

Figure S4

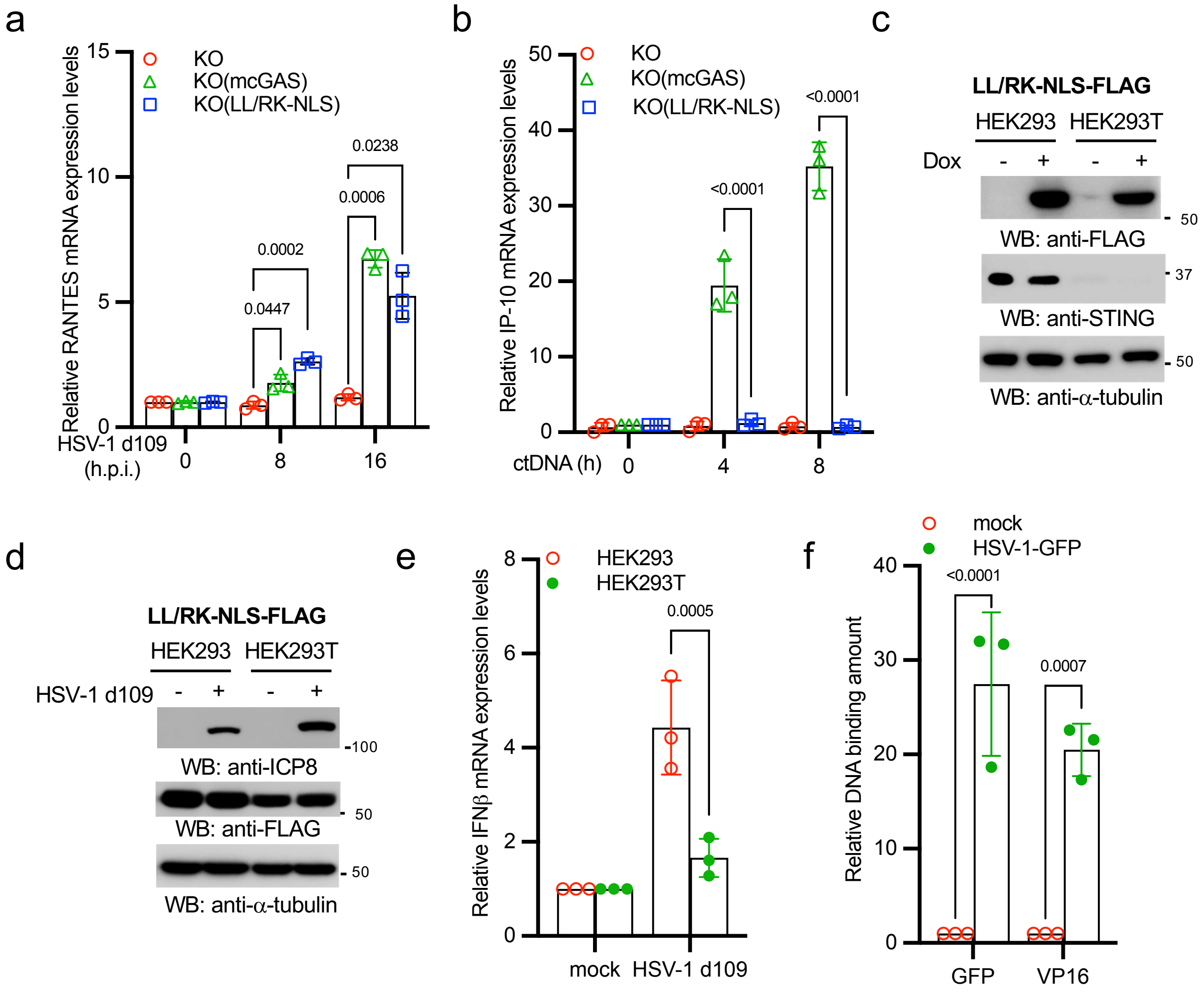
a
KO
KO(mcGAS)
KO(LL/RK-NLS)
Relative RANTES mRNA expression levels
0.0238
0.0006
0.0002
0.0447
HSV-1 d109 (h.p.i.)
0
8
16
b
KO
KO(mcGAS)
KO(LL/RK-NLS)
Relative IP-10 mRNA expression levels
<0.0001
<0.0001
ctDNA (h)
0
4
8
c
LL/RK-NLS-FLAG
HEK293
HEK293T
Dox
WB: anti-FLAG
WB: anti-STING
WB: anti-α-tubulin
d
LL/RK-NLS-FLAG
HEK293
HEK293T
HSV-1 d109
WB: anti-ICP8
WB: anti-FLAG
WB: anti-α-tubulin
e
HEK293
HEK293T
Relative IFNβ mRNA expression levels
0.0005
mock
HSV-1 d109
f
mock
HSV-1-GFP
Relative DNA binding amount
<0.0001
0.0007
GFP
VP16

Figure S5

a

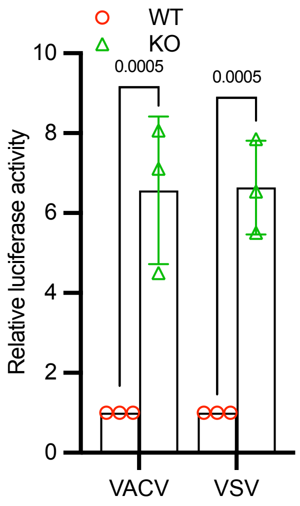

WT
KO
0.0005
0.0005
Relative luciferase activity
10
8
6
4
2
0
VACV
VSV

Figure S6

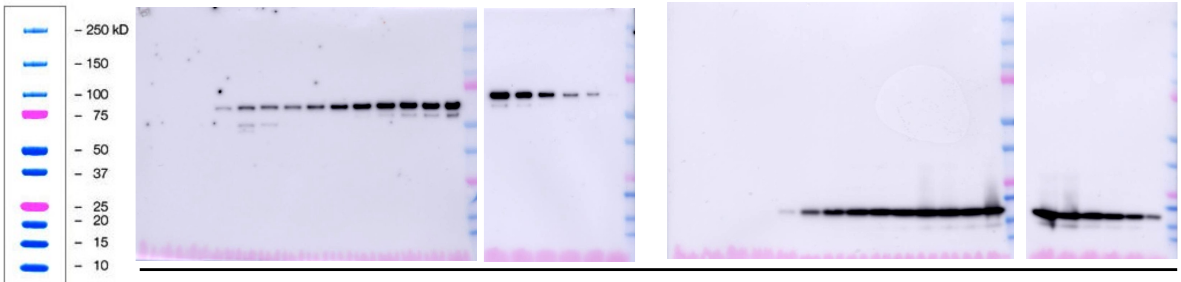

Fig. 1a

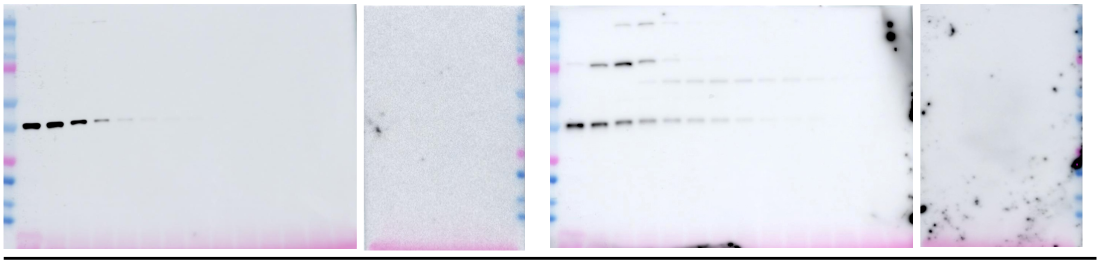

Fig. 1a

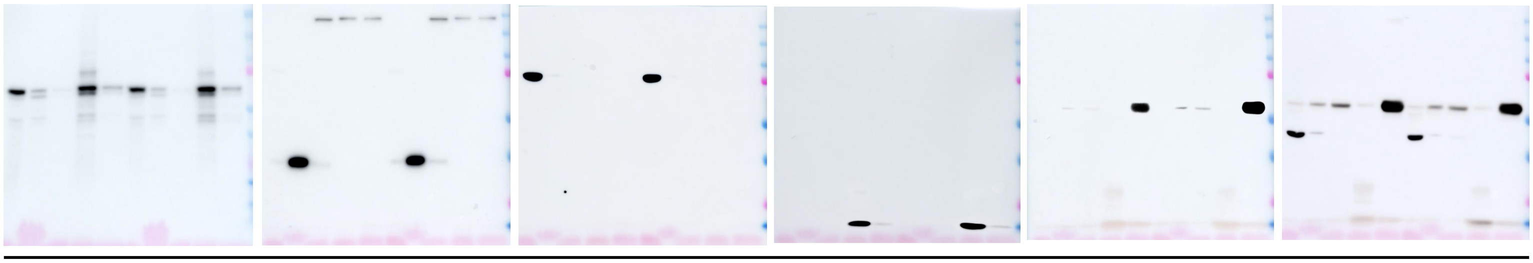

Fig. 1b

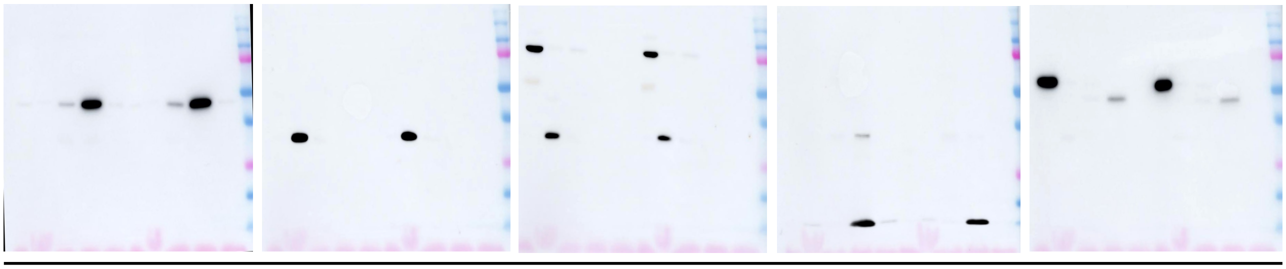

Fig. 1g

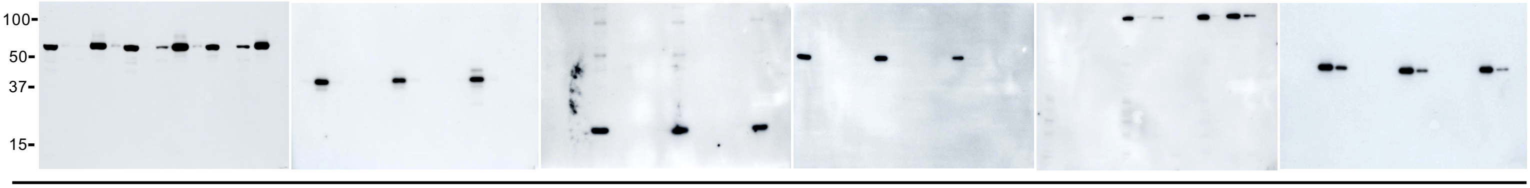

Fig. 2a

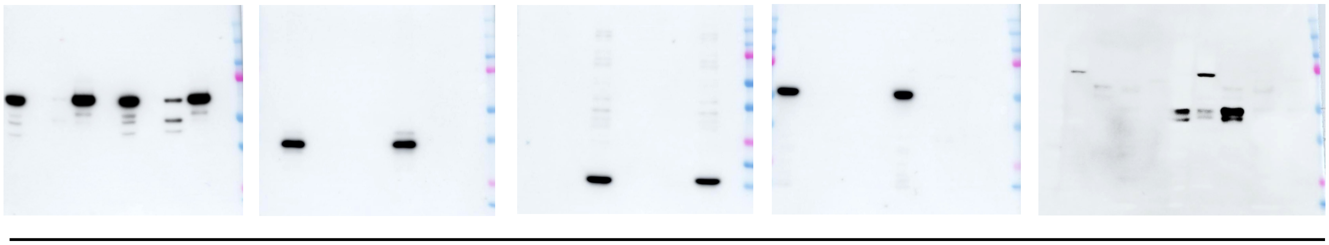

Fig. 2b

Figure S7

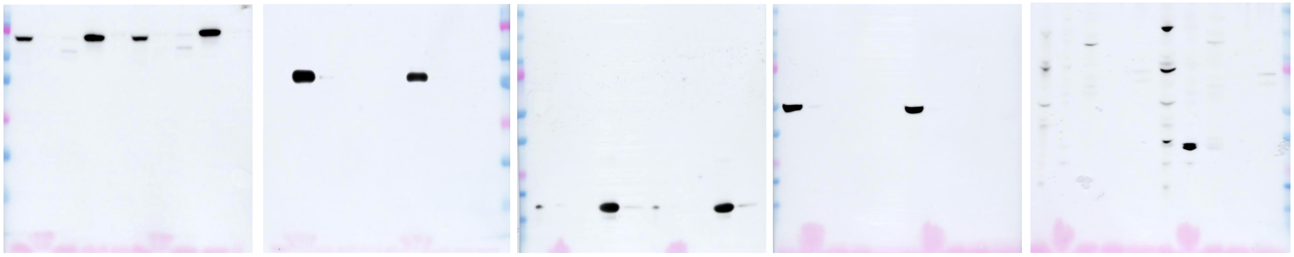

Fig. 2c

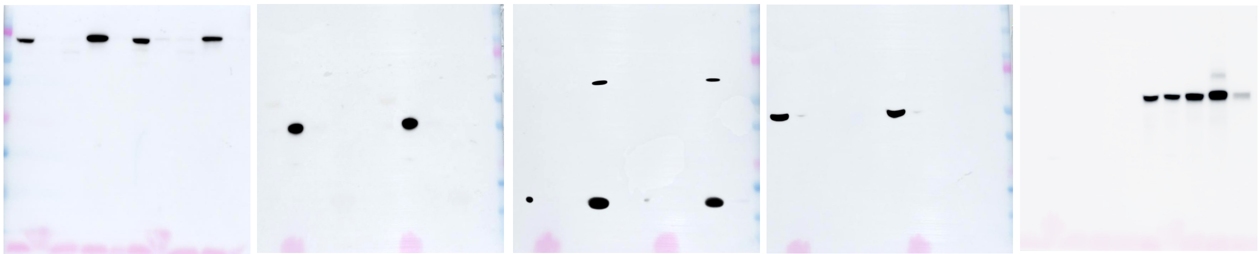

Fig. 2d

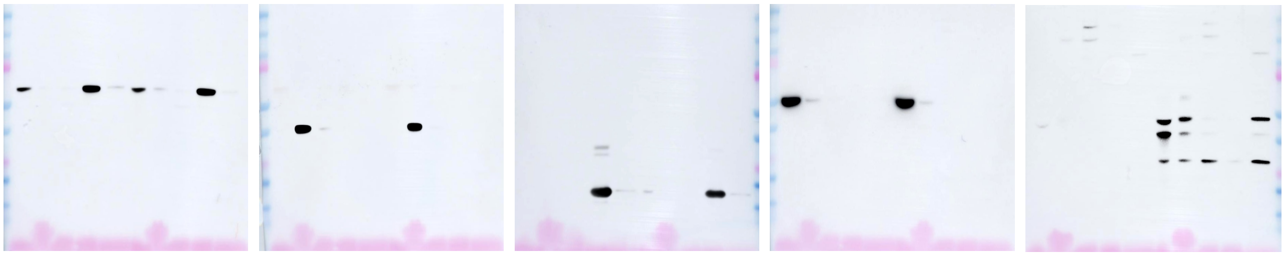

Fig. 2e

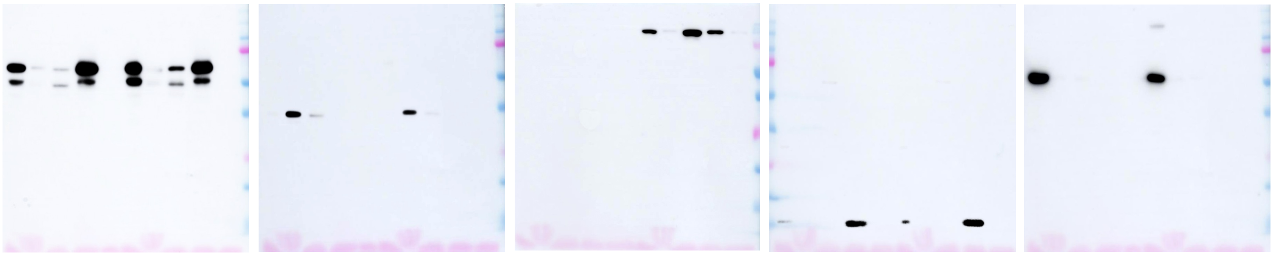

Fig. 3a

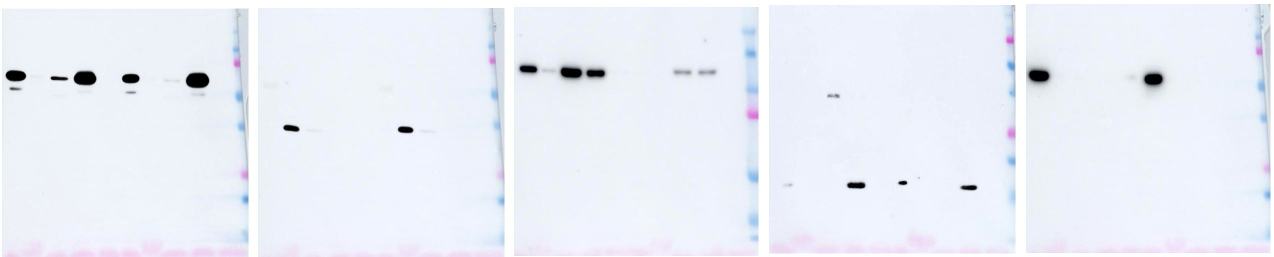

Fig. 3b

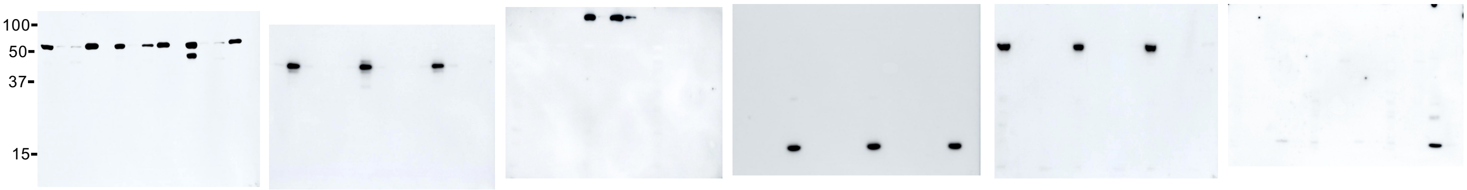

Fig. 3c

Figure S8

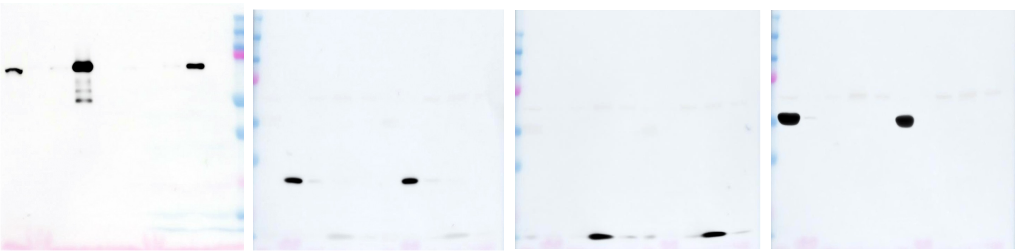

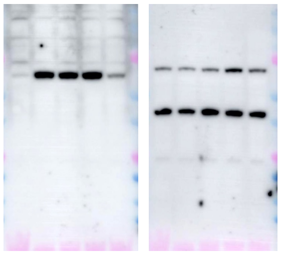

Fig. 4a

Fig. 4c

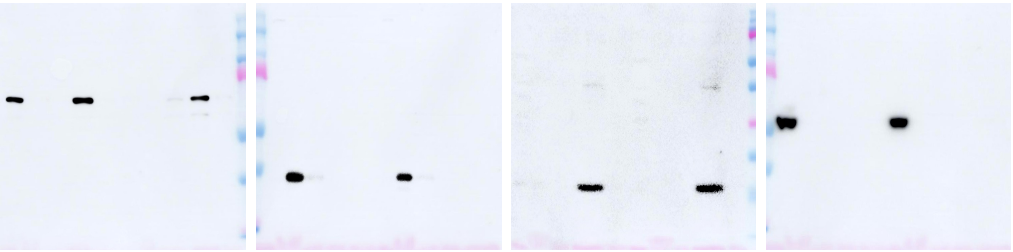

Fig. 4d

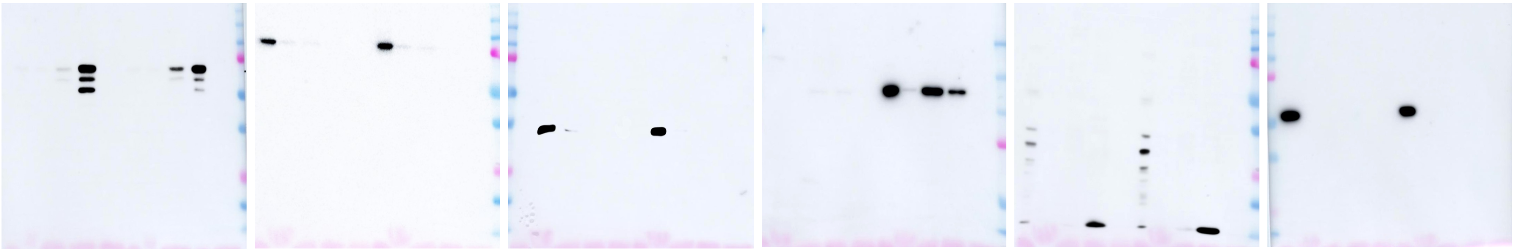

Fig. 5a

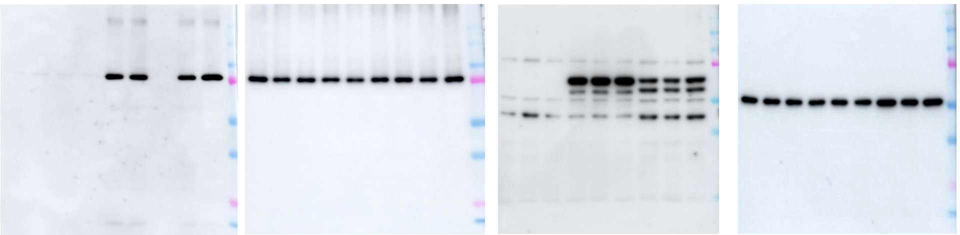

Fig. 5d

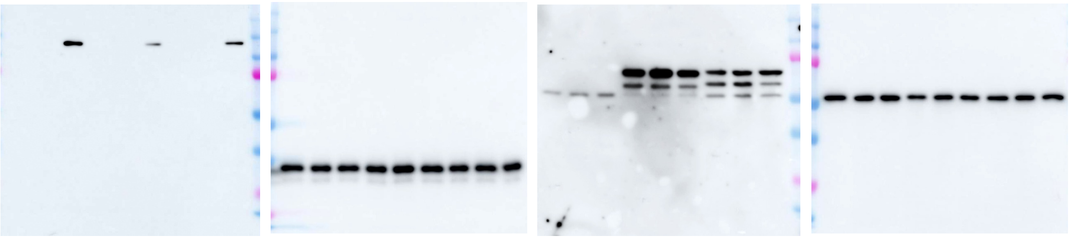

Fig. 6c

Figure S9

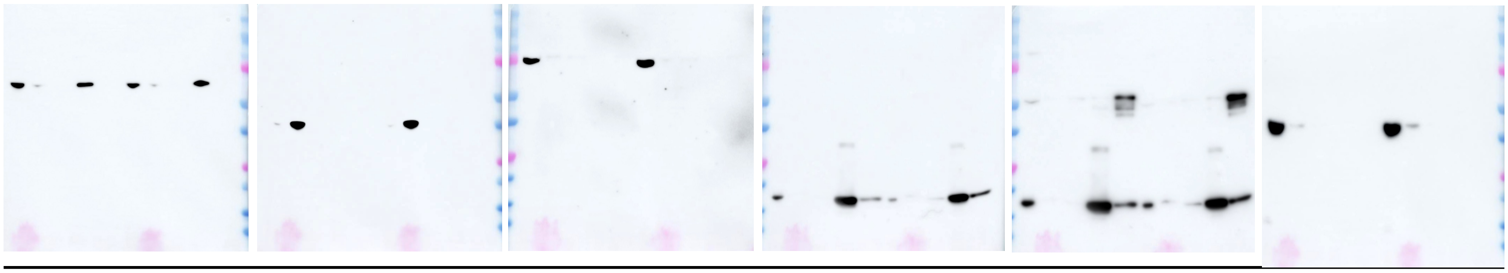

Fig. S1a

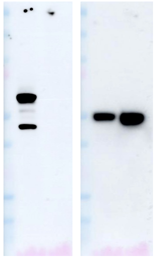

Fig. S1b

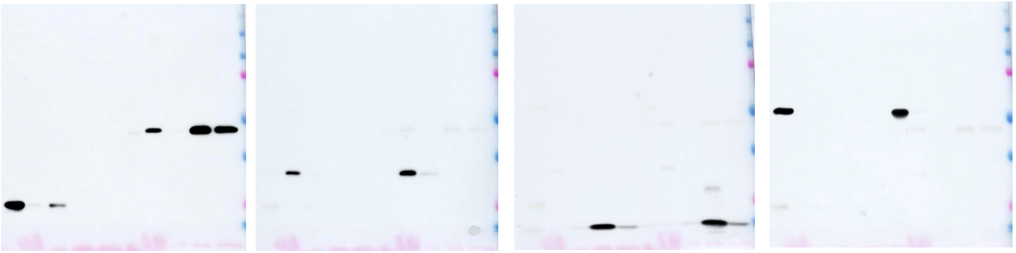

Fig. S1l

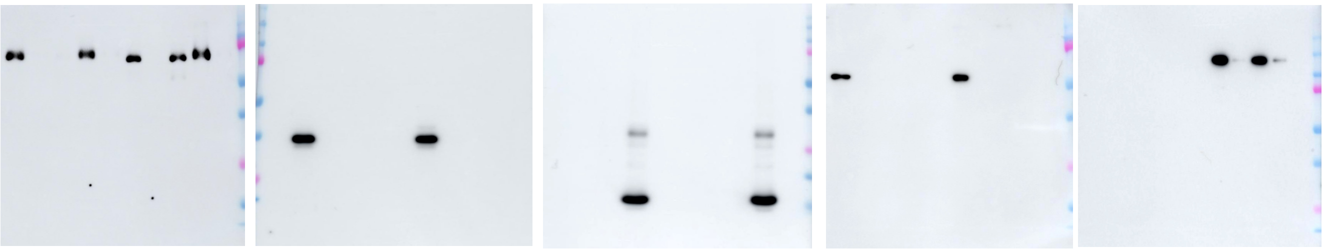

Fig. S2a

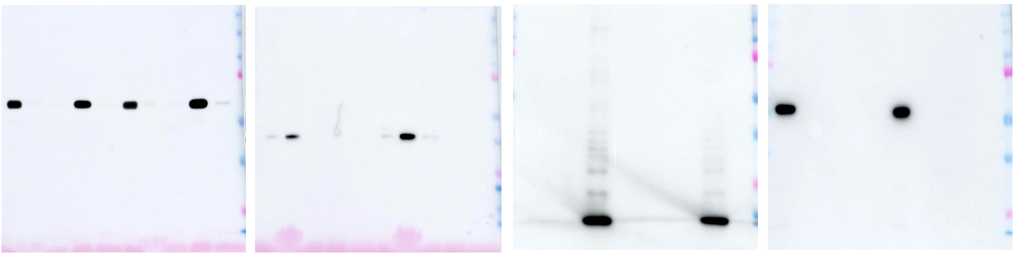

Fig. S2b

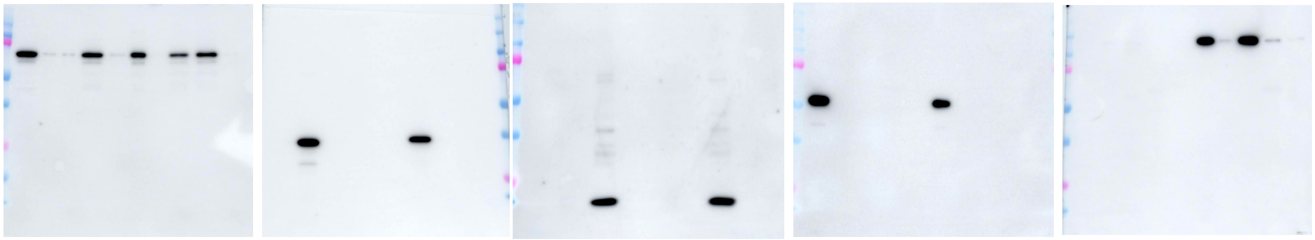

Fig. S2c

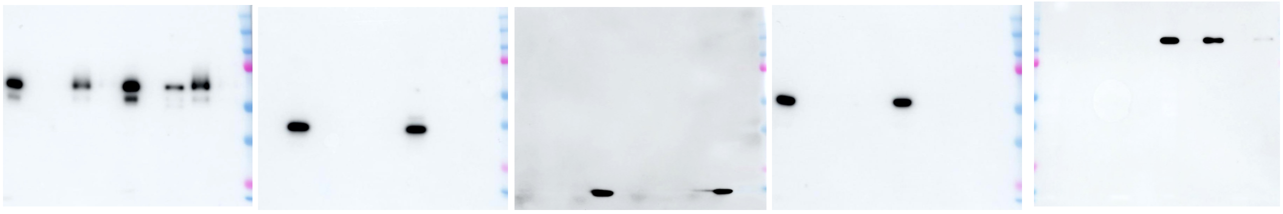

Fig. S2d

Figure 10

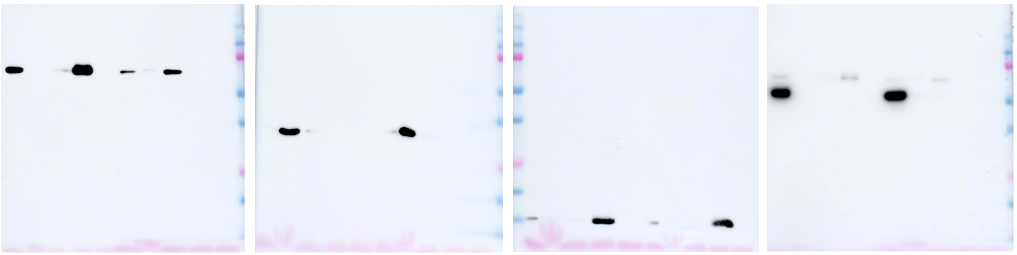

Fig. S3a

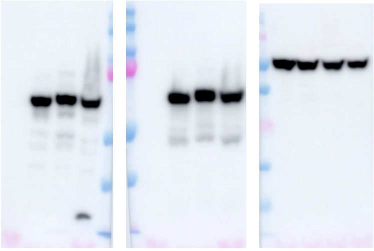

Fig. S4a

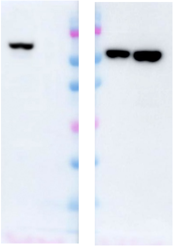

Fig. S4c

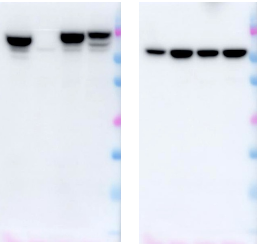

Fig. S4e

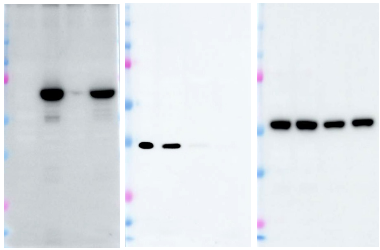

Fig. S5d

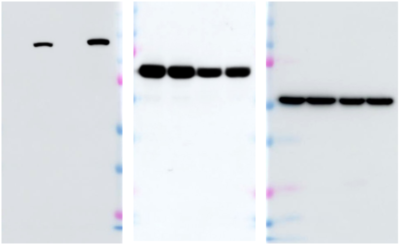

Fig. S5e

Figure S11
