# Supplementary material for: Nuclear soluble cGAS senses double-stranded DNA virus infection
Source: Commun Biol. 2022 May 10;5:433. doi: 10.1038/s42003-022-03400-1 (PMC9090744; doi:10.1038/s42003-022-03400-1)
Supplement: Supplementary file 3 — Description of Additional Supplementary Files [file 42003_2022_3400_MOESM3_ESM.pdf]

Description of Additional Supplementary Files

File name: Supplementary Data 1
Description: Source data for figures.
