# Supplementary material for: Nuclear soluble cGAS senses double-stranded DNA virus infection
Source: Commun Biol. 2022 May 10;5:433. doi: 10.1038/s42003-022-03400-1 (PMC9090744; doi:10.1038/s42003-022-03400-1)
Supplement: Supplementary file 5 — Reporting Summary [file 42003_2022_3400_MOESM5_ESM.pdf]

nature portfolio

Corresponding author(s): Shitao Li

Last updated by author(s): Apr 15, 2022

Reporting Summary

Nature Portfolio wishes to improve the reproducibility of the work that we publish. This form provides structure for consistency and transparency in reporting. For further information on Nature Portfolio policies, see our Editorial Policies and the Editorial Policy Checklist.

Statistics

For all statistical analyses, confirm that the following items are present in the figure legend, table legend, main text, or Methods section.

n/a	Confirmed	
☐	☒	The exact sample size (*n*) for each experimental group/condition, given as a discrete number and unit of measurement
☐	☒	A statement on whether measurements were taken from distinct samples or whether the same sample was measured repeatedly
☐	☒	The statistical test(s) used AND whether they are one- or two-sided<br>*Only common tests should be described solely by name; describe more complex techniques in the Methods section.*
☒	☐	A description of all covariates tested
☒	☐	A description of any assumptions or corrections, such as tests of normality and adjustment for multiple comparisons
☐	☒	A full description of the statistical parameters including central tendency (e.g. means) or other basic estimates (e.g. regression coefficient) AND variation (e.g. standard deviation) or associated estimates of uncertainty (e.g. confidence intervals)
☒	☐	For null hypothesis testing, the test statistic (e.g. *F*, *t*, *r*) with confidence intervals, effect sizes, degrees of freedom and *P* value noted<br>*Give P values as exact values whenever suitable.*
☒	☐	For Bayesian analysis, information on the choice of priors and Markov chain Monte Carlo settings
☒	☐	For hierarchical and complex designs, identification of the appropriate level for tests and full reporting of outcomes
☒	☐	Estimates of effect sizes (e.g. Cohen's *d*, Pearson's *r*), indicating how they were calculated

Our web collection on statistics for biologists contains articles on many of the points above.

Software and code

Policy information about availability of computer code

Data collection: *Provide a description of all commercial, open source and custom code used to collect the data in this study, specifying the version used OR state that no software was used.*

Data analysis: *Provide a description of all commercial, open source and custom code used to analyse the data in this study, specifying the version used OR state that no software was used.*

For manuscripts utilizing custom algorithms or software that are central to the research but not yet described in published literature, software must be made available to editors and reviewers. We strongly encourage code deposition in a community repository (e.g. GitHub). See the Nature Portfolio guidelines for submitting code & software for further information.

Data

Policy information about availability of data

All manuscripts must include a data availability statement. This statement should provide the following information, where applicable:

- Accession codes, unique identifiers, or web links for publicly available datasets
- A description of any restrictions on data availability
- For clinical datasets or third party data, please ensure that the statement adheres to our policy

The authors declare that the data supporting the findings of this study are available within the article and its Supplementary Information files, and from the corresponding author upon request.

Field-specific reporting

Please select the one below that is the best fit for your research. If you are not sure, read the appropriate sections before making your selection.

☒ Life sciences ☐ Behavioural & social sciences ☐ Ecological, evolutionary & environmental sciences

For a reference copy of the document with all sections, see nature.com/documents/nr-reporting-summary-flat.pdf

Life sciences study design

All studies must disclose on these points even when the disclosure is negative.

Sample size	The sample size was sufficient for data analysis using paired two-tailed Student's t-test. For all statistical analysis, differences were considered to be statistically significant at values of P<0.05.
Data exclusions	No data exclusions
Replication	All experiments were reproduced to reliably support conclusions stated in the manuscript.
Randomization	Samples were randomly divided into experimental groups.
Blinding	Investigators were blinded to group allocation during data collection.

Reporting for specific materials, systems and methods

We require information from authors about some types of materials, experimental systems and methods used in many studies. Here, indicate whether each material, system or method listed is relevant to your study. If you are not sure if a list item applies to your research, read the appropriate section before selecting a response.

Materials & experimental systems

n/a	Involved in the study	
☐	☒	Antibodies
☐	☒	Eukaryotic cell lines
☒	☐	Palaeontology and archaeology
☒	☐	Animals and other organisms
☒	☐	Human research participants
☒	☐	Clinical data
☒	☐	Dual use research of concern

Methods

n/a	Involved in the study	
☒	☐	ChIP-seq
☒	☐	Flow cytometry
☒	☐	MRI-based neuroimaging

Antibodies

Antibodies used	Anti-β-actin (Abcam, # ab8227), anti-FLAG (Sigma, # F3165), anti-TBK1 (Cell Signaling Technology, # 3504S], anti-phospho-TBK1 (Ser172) (Cell Signaling Technology, # 5483S), anti-C1QBP (Cell Signaling Technology, # 6502S), anti-α-Tubulin (Cell Signaling Technology, # 3873S), anti-Histone H3 (Cell Signaling Technology, # 4499S), anti-STING (Cell Signaling Technology, # 50494S), anti-vimentin (R&D Systems, MAB21052-SP), anti-ICP8 (Abcam, ab20194), anti-γ-H2AX (ABclonal, AP0099), anti-HDAC2 (Cell Signaling Technology, #5113), anti-cGAS (Cell Signaling Technology, #79978), anti-cGAS (Cell Signaling Technology, #31659).
Validation	cGAS antibodies were verified in cGAS knockout cells. All other antibodies were validated by companies and verified by the molecular weight in the SDS-PAGE gel.

Eukaryotic cell lines

Policy information about cell lines

Cell line source(s)	HEK293 cells (ATCC, # CRL-1573), RAW 264.7 (ATCC, # TIB-71), NCI-H596 cells (ATCC, HTB-178), HFF-1 (ATCC, #SCRC-1041), L929 (ATCC, # CRL-6364), MDA-MB-231 (Sigma, 92020424-1VL), Vero cells (ATCC, # CCL-81), NCI-H1299 cells (ATCC, # CRL-5083) and THP-1 cells (ATCC, TIB-202).
Authentication	All cell lines have been thoroughly tested and authenticated by ATCC
Mycoplasma contamination	All cell lines are tested Mycoplasma negative by MycoAlert™ Mycoplasma Detection Kit from Lonza.
Commonly misidentified lines (See ICLAC register)	*Name any commonly misidentified cell lines used in the study and provide a rationale for their use.*
